# Nanodrugs Detonate Lysosome Bombs

**DOI:** 10.3389/fphar.2022.909504

**Published:** 2022-05-17

**Authors:** Yuting Xiang, Niansheng Li, Min Liu, Qiaohui Chen, Xingyu Long, Yuqi Yang, Zuoxiu Xiao, Jia Huang, Xiaoyuan Wang, Yunrong Yang, Jinping Zhang, Chong Liu, Qiong Huang

**Affiliations:** ^1^ Xiangya School of Pharmaceutical Sciences, Central South University, Changsha, China; ^2^ Department of Pharmacy, Xiangya Hospital, Central South University, Changsha, China; ^3^ National Clinical Research Center for Geriatric Disorders, Xiangya Hospital, Central South University, Changsha, China; ^4^ Hunan Provincial Key Laboratory of Cardiovascular Research, Xiangya School of Pharmaceutical Sciences, Central South University, Changsha, China; ^5^ Departments of Clinical Pharmacology and Pharmacy, Hunan Key Laboratory of Pharmacogenetics, National Clinical Research Center for Geriatric Disorders, Xiangya Hospital, Central South University, Changsha, China; ^6^ Institute of Clinical Pharmacology, Engineering Research Center of Applied Technology of Pharmacogenomics, Ministry of Education, Central South University, Changsha, China

**Keywords:** lysosomal membrane permeabilization, nanomaterials, nanodrugs, cancer treatment, chemodynamic therapy, magnetic nanoparticles, subcellular organelle-targeting

## Abstract

Cancer cell lysosomes contain various hydrolases and non-degraded substrates that are corrosive enough to destroy cancer cells. However, many traditional small molecule drugs targeting lysosomes have strong side effects because they cannot effectively differentiate between normal and cancer cells. Most lysosome-based research has focused on inducing mild lysosomal membrane permeabilization (LMP) to release anticancer drugs from lysosomal traps into the cancer cell cytoplasm. In fact, lysosomes are particularly powerful “bombs”. Achieving cancer cell-selective LMP induction may yield high-efficiency anticancer effects and extremely low side effects. Nanodrugs have diverse and combinable properties and can be specifically designed to selectively induce LMP in cancer cells by taking advantage of the differences between cancer cells and normal cells. Although nanodrugs-induced LMP has made great progress recently, related reviews remain rare. Herein, we first comprehensively summarize the advances in nanodrugs-induced LMP. Next, we describe the different nanodrugs-induced LMP strategies, namely nanoparticles aggregation-induced LMP, chemodynamic therapy (CDT)-induced LMP, and magnetic field-induced LMP. Finally, we analyze the prospect of nanodrugs-induced LMP and the challenges to overcome. We believe this review provides a unique perspective and inspiration for designing lysosome-targeting drugs.

## Introduction

Cancer burdens global public health; it accounts for nearly 10 million deaths annually worldwide, and this number keeps increasing (Sung et al., 2021). The battle against tumors has been ongoing for decades, and a multitude of anticancer drugs have been developed. Many antitumor therapeutic strategies rely on the activation of the apoptosis pathway in tumor cells (Kolenko et al., 2000; Huang et al., 2022), a signaling cascade involving caspases leading to apoptosis (also known as caspase-dependent cell death). However, tumor cells are resistant to apoptosis-inducing and caspase-activating drugs by mutating pro-apoptotic proteins and overexpressing anti-apoptotic proteins (Giampazolias et al., 2017). Interestingly, some cellular components may achieve self-killing by acting as “bombs” (Zhang et al., 2021).

Lysosomes, discovered in 1955, have long been regarded as mere “digesting” machines (De Duve and Wattiaux, 1966). These organelles are essential for endocytosis and autophagy (Piao and Amaravadi, 2016) and contain numerous hydrolases (De Duve and Wattiaux, 1966) responsible for degrading and recycling macromolecules such as exogenous substances and intracellular metabolic waste. In addition, lysosomes play a key role in many cellular biochemical processes, such as cell death and signal transduction (Platt, 2018). Severely damaged lysosomal membranes release the highly active hydrolases from the lysosome into the cytoplasm, where they affect various cellular components and activate death pathways. This process is known as lysosomal membrane permeabilization (LMP)-mediated lysosomal cell death (Kirkegaard and Jäättelä, 2009). The role of LMP in the cell death process is complex and involves various cell death pathways such as apoptosis, necrosis, and pyroptosis (Repnik et al., 2014). In short, leaky and ruptured lysosomes can become bombs threatening cell survival by releasing self-killing enzymes and impairing cellular functions.

These lysosomal bombs have also been called “suicide bags” (De Duve and Wattiaux, 1966). Besides their immense potential power, lysosomes are crucial for the rapid division of tumors; therefore, they have received extensive attention from tumor treatment researchers. Transformation and cancer progression involve dramatic changes in lysosomal volume, composition, and cellular distribution (Serrano-Puebla and Boya, 2018). In tumor cells, lysosomes are hypertrophic and more abundant (Galluzzi et al., 2018) and, therefore, more fragile and prone to rupture than in normal cells (Ma et al., 2020). Moreover, tumor cells have higher lysosomal enzymes levels (Serrano-Puebla and Boya, 2018). For example, increased expression of cathepsin B has been proposed as a marker for colorectal cancer (Abdulla et al., 2017), and various tumors express high cathepsin D levels (Fukuda et al., 2005). The morphological and physiological changes of lysosomes in tumor cells favor LMP induction. Some “detonators”—such as detergents or lipophilic drugs (Zhitomirsky and Assaraf, 2016)—can accumulate in lysosomes and induce LMP. For example, cationic amphoteric drugs induced LMP-mediated cell death by detaching acid sphingomyelinase from lysosomal membranes (Serrano-Puebla and Boya, 2018). However, these drugs may not be designed for LMP induction and lysosomal cell death pathways activation, and most of them have limitations such as low tumor selectivity and low lysosomal specificity.

**FIGURE 1:**
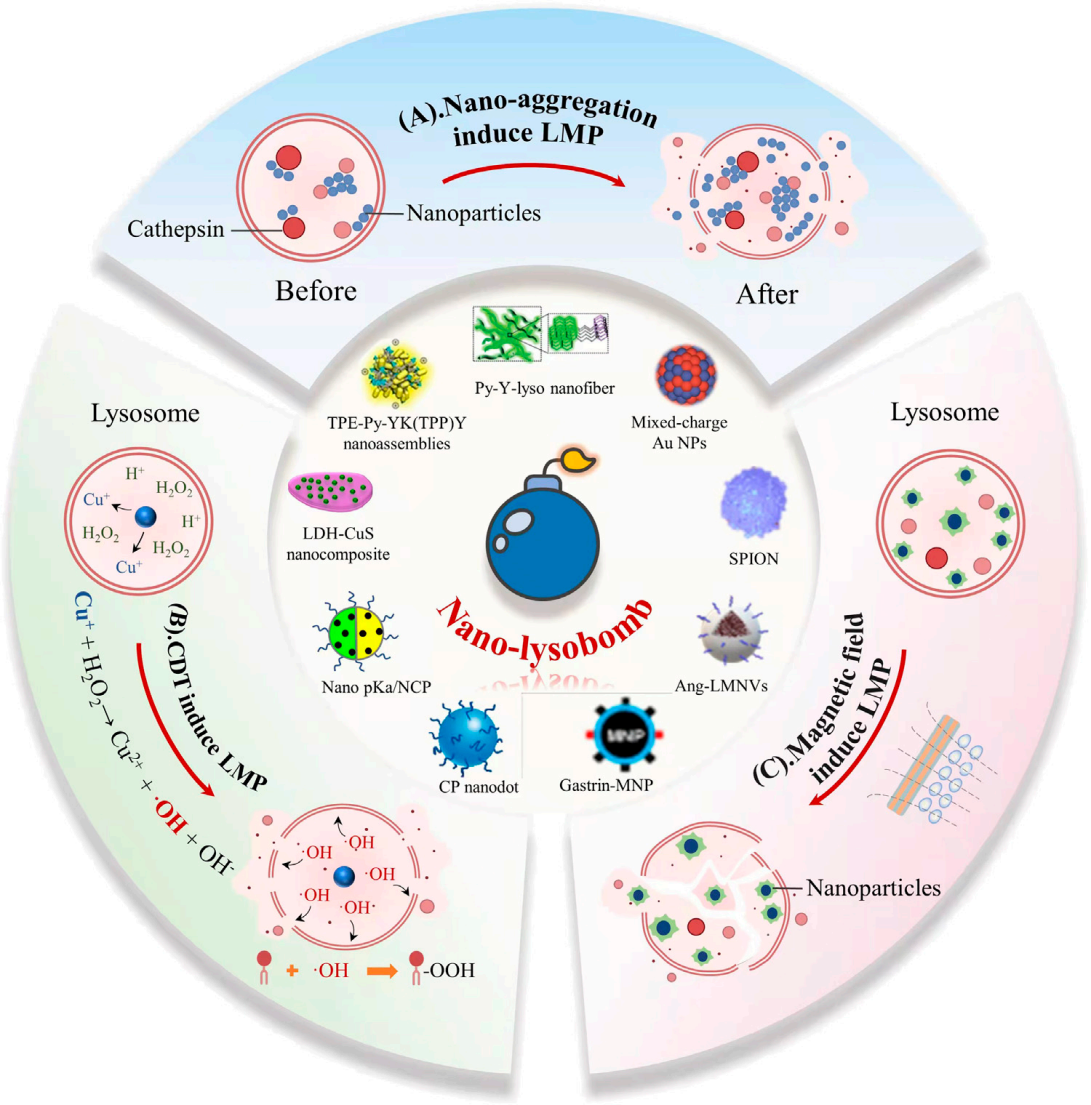
Schematic illustration of nano-lysobombs inducing LMP and lysosome cell death. Nano-lysobombs are classified into three groups according to their action mechanism: nano-aggregation, CDT process, and magnetic field induction **(A)** The first class of nanodrugs triggers osmotic flow and lysosome swelling by self-assembling on the cell membrane or aggregating in lysosomes **(B)** The second class of nanodrugs triggers the Fenton reaction in the lysosomes, using generated OH to induce lipid peroxidation (LPO) of the lysosomal membrane and then trigger LMP **(C)** The third class uses the thermal effects or mechanical stress generated by magnetic field-stimulated magnetic nanoparticles to destroy the lysosomal membranes, eventually killing the cells.

The tremendous progress in nanodrugs has enabled the discovery of novel effective “detonators” to ignite lysosome bombs in tumor cells. First, most cellular uptake pathways of nanomedicines mediate endocytosis into lysosomes, including phagocytosis, clathrin-mediated endocytosis, and macropinocytosis (Stern et al., 2012), making this organelle the most common intracellular site of nanoparticle sequestration and degradation. Taking advantage of this, most LMP-inducing nanodrugs can reach their target (lysosomes) with little off-target effects. Second, the enhanced permeability and retention effect (EPR effect) (Kanamala et al., 2016) in tumors allows nanodrugs to achieve high tumor selectivity. In addition, modifying the nanodrugs with functional groups that specifically recognize cancer cells or respond to the tumor microenvironment increases their selectivity (Wang et al., 2021; Yang et al., 2022). Finally, nanodrugs have flexible physicochemical properties, and those with reasonable components and specific functional design can cleave lysosomes more efficiently than small molecule drugs, yielding more potent anticancer effects (Ai et al., 2021). There are growing interests and attempts to design “detonators” of lysosome bombs combining the advantages of nanodrugs (nano-lysobombs). Recently, some anticancer nano-lysobombs have been developed and have demonstrated potent antitumor effects and few side effects. However, to our knowledge, no review summarizes the newly developed anticancer nano-lysobombs. Thus, this minireview discusses the promising nano-lysobombs initiating LMP and lysosomal cell death in tumors. These nano-lysobombs are categorized into three groups based on their initiation process: nano-aggregation, chemodynamic therapy (CDT) and magnetic field induction (Figure 1, Table 1). Moreover, we detail the advantages and disadvantages of nano-lysobombs to highlight their potential.

**TABLE 1:** Nanodrugs detonate lysobombs.

Classification	Nanomaterials	Size	Targeting Strategy	Sources
Nano-aggregation-induced LMP	[+/−] NPs	5.3 ± 0.7 nm of Au NPs ≈200 nm aggregates	Mixed-charge modification for lysosomal targeting	Borkowska et al. (2020)
	Py-Yp-lyso	74.6 ± 11.6 nm aggregates	Alkaline phosphatase guides for tumor targeting; 4-(2-aminoethyl) morpholine for lysosomal targeting	Wu et al. (2021)
	TPE-Py-pYK(TPP)pY	310 nm of aggregates	Alkaline phosphatase guides for tumor targeting; Proper surface charge and nanosize for lysosomal endocytosis	Ji et al. (2021)
CDT-induced LMP	LDH-CuS NCs	≈120 nm (TEM)	LDH plate-like morphology for lysosomal endocytosis	Liu et al. (2020)
	CP nanodot	≈5.0 nm (TEM) ≈ 16.3 nm (hydrodynamic diameter)	—	Lin et al. (2019)
	nano pKa/NCP	≈90 nm (TEM) ≈ 100 nm (hydrodynamic diameter)	—	Deng et al. (2022)
Magnetic field-induced LMP	SPIONs	≈60 nm (hydrodynamic diameter)	—	Lunov et al. (2019)
	Ang—LMNVs	≈179 ± 3 nm (hydrodynamic diameter)	Angiopep-2 guides for tumor targeting	Pucci et al. (2020)
	Gastrin-MNP	≈37–50 nm	Gastrin guides for tumor targeting	Clerc et al. (2018)

## Nano-Aggregation-Induced Lysosomal Membrane Permeabilization

Cancer cells present an altered lysosomal membrane structure making them more susceptible to osmotic swelling (Ma et al., 2020). Therefore, the idea of constructing nanomedicines inducing lysosome osmotic pressure difference or causing lysosome morphological changes has emerged, and nano-aggregation strategies have been developed. After reaching the plasma membrane of cancer cells, the nanoparticles would first enter early endosomes and then be transported to late endosomes—also called multivesicular bodies (MVB) (Hessvik and Llorente, 2018; Beatriz et al., 2021)—which then fuse with lysosomes. If these specially designed nanoparticles aggregate or self-assemble to a large scale at any stage of internalization, LMP will be induced through ion-induced osmotic pressure and lysosomal swelling, subsequent cancer cell death (Baehr et al., 2021; Wang et al., 2022).

The nano-aggregation strategies aim to selectively target cancer cells to achieve an excellent anticancer effect with few side effects. Currently, two nano-aggregation-induced LMP strategies exist. The first strategy (Borkowska et al., 2020) is based on the difference in lysosomal pH between cancer cells and normal cells. Cancer cell lysosomes and MVBs have lower pH (pH lysosome ≈4.2 in MDA-MB-231 cells) (Borkowska et al., 2020) than normal cell lysosomes (pH ≈ 4.8) and multilamellar bodies (pH < 6.1) (Lajoie et al., 2005). Recently, Borkowska et al. (Borkowska et al., 2020) developed a series of Au nanoparticles [(+/−) NPs] modified with the positively charged *N*,*N*,*N*-trimethyl (11-mercaptoundecyl) ammonium chloride (TMA) and the negatively charged 11-mercaptoundecanoic acid (MUA) at different ratios to specifically induce LMP in cancer cells. A decrease in pH protonates the carboxyl group of the MUA and decreases the negative charge, rendering MUA hydrophobic. Interestingly (+/−) NPs with a TMA/MUA ratio of 80:20 only aggregated and triggered LMP in the lysosomes of cancer cells, but not in multilamellar bodies of normal cells. These (+/−) NPs (χTMA:χMUA = 80:20) had a particle size of only 5.3 nm and formed very large aggregates with a diameter >2 μm in cancer lysosomes, ultimately resulting in a powerful and broad-spectrum LMP-induced anticancer effect. Furthermore, in normal cells, the 80:20 (+/−) NPs were only present in multilamellar bodies, and most of them were excreted by exocytosis, indicating the potentially low adverse effects.

The second strategy (Ji et al., 2021; Wu et al., 2021) employs an enzyme-instructed self-assembly (EISA) approach. Alkaline phosphatases are highly expressed on the plasma membrane of many types of cancer cells and have been adopted as ELSA-promoting enzymes to induce LMP by mediating nano-aggregation (Wang et al., 2016; Gao et al., 2020a). For example, Wu et al. (Wu et al., 2021) constructed Py-Phe-Phe-Glu-Tyr (H_2_PO_3_)-Gly-Lyso (Py-Yp-Lyso) to treat cervical cancer through nano-aggregation-inducing LMP. Py-Yp-Lyso consisted of four parts: Glu-Tyr (H_2_PO_3_) was dephosphorylated by alkaline phosphatases to form Py-Y-Lyso and further self-assembled into nanofibers. The pyrene molecule (Py) served as a monitor due to its concentration-dependent luminescence. Phe-Phe conferred better biostability as a self-assembling structure. Finally, the 4-(2-aminoethyl) morpholine served as a lysosome targeting molecule (Lyso). It is prone to protonation, becomes more hydrophilic into the acidic lysosome, and remains there instead of diffusing to the cytoplasm. Py-Yp-Lyso had a negative charge and was stable in the blood circulation system because it contained a phosphate group. When Py-Yp-Lyso reached the cancer cells, alkaline phosphatases on the surface of the cancer cell plasma membrane dephosphorylated it, reversing its charge. The positively charged surface of the dephosphorylated Py-Y-Lyso promoted endocytosis and protonated the 4-(2-aminoethyl) morpholine moiety, preventing Py-Y-Lyso from diffusing out of the lysosome. In acidic lysosomes, Py-Yp-Lyso aggregation formed large nanofibers which triggered LMP. Py-Yp-Lyso had significantly higher cytotoxicity in Hela cells than in normal cells and inhibited tumor growth to 55.4% of that of control tumors at day 15 following the injection. Similarly, Ji et al. (Ji et al., 2021) constructed an LMP trigger called TPE-Py-pYK(TPP)pY, which was composed of three segments: tetraphenylethylene-pyride (TPE-Py) mainly for imaging, pYKpY (pY = phosphotyrosine) for self-assembly, and triphenylphosphine (TPP) for charge regulation and internalization. Treating Hela cells with TPE-Py-pYK(TPP)pY caused lysosomal destruction and high cytotoxicity. Intriguingly, LMP also effectively induced immunogenic cell death and ameliorated the immunosuppressive microenvironment.

## CDT-Induced Lysosomal Membrane Permeabilization

Reactive oxygen species (ROS) are highly reactive and, at high concentrations, they can react with unsaturated lipids in lysosomal membranes to trigger LMP (Huang et al., 2017). CDT kills cancer cells by converting the high concentrations of endogenous H_2_O_2_ in cancer cells into the highly oxidative OH through the Fenton reaction (Chen et al., 2022). CDT has very excellent selectivity and causes few side effects because tumors have much higher H_2_O_2_ concentrations than normal tissue (Tang et al., 2019). The CDT reaction requires a low pH (pH ≈ 2–4). However, many Fenton reagents exhibit low efficiency in the weakly acidic tumor microenvironment (pH ≈ 6) (Shi et al., 2021) or cytoplasm (pH > 7.2) (Persi et al., 2018), prompting researchers (Dong et al., 2020; Shi et al., 2021) to search for a more favorable acidic environment for CDT and develop Fenton reagents with broader reaction conditions. The lysosome provides a favorable acidic environment (pH ≈ 4–5) for efficient CDT, and the Fenton reaction exhibits a higher reaction rate in such an acidic environment (Zhao et al., 2019). Moreover, lysosomes rarely contain antioxidant enzymes that inhibit the efficacy of CDT (such as catalase, glutathione peroxidase) (Kurz et al., 2008) but do enclose some small biomolecules that benefit CDT (cysteine, ascorbic acid, and glutathione for reducing oxidated Fenton reagents) (Kurz et al., 2008). Combining the high H_2_O_2_ levels in tumors and favorable conditions for the Fenton reaction in lysosomes, CDT-induced LMP is highly efficient and has a potent anticancer effect.

Copper-based nanoparticles can be used in a wide range of acidic environments (Jia et al., 2022) and exhibit a more efficient catalytic rate (Zhao et al., 2020) as a Fenton reaction catalyst than traditional ferrous materials. Therefore, copper-based nanomaterials are wildly used in CDT-induced LMP. Recently, Liu et al.(Liu et al., 2020) layered double hydroxide-copper sulfide nanocomposites (LDH-CuS NCs) to effectively treat cancer by CDT-induced LMP. They prepared LDH-CuS NCs by *in situ* growth of CuS on LDH nanosheets. The group chose CuS nanodots for their photothermal activity and better biosafety and performed near-infrared (NIR) excitation to release thermal effects-inducing copper ions to initiate Fenton-like reactions. This process is called NIR-induced CDT. Moreover, the sheet-like structure of LDH allowed CuS to stay in or adhere to lysosomes for longer periods, as 1D and 2D materials remain longer in endocytosis than spherical nanoparticles do. Therefore, LDH-CuS exhibited strong lysosomal localization ability, while free CuS nanodots are prone to diffuse into the cytoplasm. Finally, NIR-excited LDH-CuS efficiently generated OH in lysosomes, inducing LMP and achieving complete tumor suppression, while free CuS nanodots had poor tumor-suppressive effects, demonstrating the importance of lysosomes in CDT.

A continuous H_2_O_2_ supply is essential to maintain CDT and the subsequent LMP process. The exhaustion of endogenous H_2_O_2_ in lysosomes of tumor cells hampers CDT efficacy (Jia et al., 2022). To overcome this limitation, Lin et al.(Lin et al., 2019) prepared copper peroxide nanodots (CP nanodots) by combining Cu^2+^ and H_2_O_2_ in the presence of poly (vinylpyrrolidone) (PVP) as a stabilizer. These nanodots simultaneously release Cu^2+^ and H_2_O_2_ in the acidic environment, especially in the lysosomes of tumor cells. CP nanodots significantly inhibited the tumor growth of U87MG tumor-bearing mice *via* CDT-inducing LMP. To further explore the retention behavior of copper-based nanomaterials in lysosomes, Deng et al. (Deng et al., 2022) prepared a series of copper peroxide nanodrugs with different pKa (Nano pKa/NCP). NCPs with low pKa (5.2–6.2) remained in lysosomes for longer and in higher quantities than those with high pKa and increased the ROS levels and LMP in lysosomes. Nano-pKa5.2/NCP treatment displayed the most apparent tumor growth suppression *in vivo*.

## Magnetic Field-Induced Lysosomal Membrane Permeabilization

Cancer treatments based on alternating magnetic fields have numerous advantages over other methods. For example, magnetic fields do not have adverse effects on the human body and have unlimited tissue penetration depth. Many antitumor magnetic nanoparticles (MNPs) have been developed, especially those made from iron oxides, since they have favorable biocompatibility, abundant raw materials, and outstanding biological effects (Soetaert et al., 2020). The MNPs vibrate violently under the alternating magnetic field, which not only induces strong mechanical stress (Lopez et al., 2022) but also increases the temperature near the MNPs (Gobbo et al., 2015). Besides, lysosomal membranes can be disrupted by mechanical stress and thermal expansion, making alternating magnetic field-induced LMP a promising approach for cancer therapy. For example, Lunov et al.(Lunov et al., 2019) experimented with magnetic stimulation-induced LMP through superparamagnetic iron oxide nanoparticles (SPIONs) against liver cancer. Carboxydextran-coated SPIONs passively accumulated in the liver and were efficiently endocytosed into lysosomes by liver cancer cells. SPIONs strongly damaged lysosomes through magneto-mechanical stress with a force ≥700 pN, induced cathepsin B leakage, and eventually killed cells exposed to high-intensity (up to 8 T), short-pulse-width (≈15 µs) pulsed magnetic field.

Pucci et al. (Pucci et al., 2020) also constructed Angiopep-2 functionalized lipid-based magnetic nanovectors (Ang-LMNVs) for magnetic field-induced LMP against glioblastoma multiforme. They added angiopep-2 on LMNVs to target the low-density lipoprotein receptor-related protein 1 (LRP1, overexpressed on the glioma cell surface) and promote blood-brain barrier penetration. Submitting the Ang-LMNVs to an alternating magnetic field induced LMP and glioma cell death through magnetic intra-lysosomal hyperthermia (MILH). Compared with magnetic hyperthermia therapy (which elevates the overall temperature of the tumor), MILH showed better biosafety by only heating the nanoparticles inside the lysosomes and was more energy-efficient (Pucci et al., 2020). In addition, Ang-LMNVs showed prior delivery efficacy and synergistic effects as nanovectors for the nongenotoxic drug nutlin-3a. Notably, Ang-LMNVs submitted to an alternating magnetic field could raise the local temperature to 43°C without affecting heat shock protein 70 (HSP70), bypassing the protection mechanism of HSP70 against magnetocaloric LMP-induced cell death.

Very recently, Clerc et al. (Clerc et al., 2018) constructed gastrin-modified iron oxide magnetic nanoparticles (Gastrin-MNPs) and confirmed their selective cytotoxicity towards various tumors. Gastrin-MNPs targeted tumor cells by recognizing overexpressed gastrin receptors (CCK2R) and inducing LMP through MILH. The MILH-induced local heating increased the ROS level by 7-fold by enhancing the Fenton reaction within lysosomes, in which iron from MNPs was partially involved.

## Conclusion and Prospects

In this review, we summarized the three main emerging nanodrug-induced LMP strategies. These nanodrugs demonstrated potent antitumor effects and had ultra-low side effects. Nevertheless, nanodrug-based lysosomal bombs still pose some problems that need to be addressed. For example, HSP70 is overexpressed in malignant tumors and is associated with poor therapeutic outcomes (Kroemer and Jäättelä, 2005; Pucci et al., 2020). HSP70 is localized in the lysosomal membrane and can protect the lysosomal membrane from different stimuli (Kirkegaard and Jäättelä, 2009), including ROS-induced LMP. This suggests that nanodrug-induced LMP researchers should pay particular attention to the factors protecting tumor cells from lysosomal cell death. In addition, the selectivity of nanodrug-induced LMP requires further improvement. For example, EISA-based strategies remain uncommon because alkaline phosphatases are not significantly expressed in some cancer cells (Li et al., 2017). Furthermore, CDT-induced LMP strategies do not differentiate between cancerous and inflamed tissues because many inflammation sites also have elevated H_2_O_2_ concentrations (Chen et al., 2021; Liu et al., 2022; Zhao et al., 2022; Zhu et al., 2022). Finally, while magnetic field-induced LMP has good spatial selectivity–because the direction of the magnetic field determines the treatment site—it leaves undetected cancer sites untreated.

LMP is a critical process affecting lysosomal function and is an intermediate link in many therapeutic strategies. Multidrug resistance is closely related to lysosomes, as lysosome chelation traps many drugs, rendering them ineffective. Therefore, numerous studies have explored mild LMP induction as a method to mediate lysosomal escape of drugs and thereby increase antitumor efficacy. In fact, due to the highly toxic compounds in lysosomes and their essential role in cellular physiology, strong LMP can directly kill tumors. However, the key to LMP-based therapy is to effectively distinguish cancer cells from normal cells, which dramatically limits the application of this otherwise extremely promising therapy. Fortunately, the many unique and flexible physicochemical properties of nanodrugs allow for specially designed nanodrugs to selectively induce strong LMP in cancer cells, which this minireview highlights. Many commonly used antitumor strategies have excellent efficiency combined with lysosomal targeting. For example, lysosomal targeting combined with photodynamic therapy (PDT) to overcome the dark toxicity of many photosensitizers (Gao et al., 2020b). To the best of our knowledge, these advantages have not yet been used for LMP-induced cell death in tumor treatment, which may be related to the need for local lighting accuracy. Nevertheless, it provides a research direction for more efficient therapies.

In general, therapeutic strategies that utilize the characteristics of lysosomes in tumor cells are efficient. In addition, the lysosomal cell death mechanism is different from that of most antitumor drugs and provides more opportunities for coping with drug resistance. In addition, processes such as development and aging may be related to the instability of lysosomal membranes. Therefore, LMP-inducing strategies can not only be applied to tumors but may also be worth studying in other aspects leading to treatments for other diseases.
